# Novel Associations of Nonstructural Loci with Paraoxonase Activity

**DOI:** 10.1155/2012/189681

**Published:** 2012-04-17

**Authors:** Ellen E. Quillen, David L. Rainwater, Thomas D. Dyer, Melanie A. Carless, Joanne E. Curran, Matthew P. Johnson, Harald H. H. Göring, Shelley A. Cole, Sue Rutherford, Jean W. MacCluer, Eric K. Moses, John Blangero, Laura Almasy, Michael C. Mahaney

**Affiliations:** Department of Genetics, Texas Biomedical Research Institute, P.O. Box 760549, San Antonio, TX 78245-0549, USA

## Abstract

The high-density-lipoprotein-(HDL-) associated esterase paraoxonase 1 (PON1) is a likely contributor to the antioxidant and antiatherosclerotic capabilities of HDL. Two nonsynonymous mutations in the structural gene, *PON1*, have been associated with variation in activity levels, but substantial interindividual differences remain unexplained and are greatest for substrates other than the eponymous paraoxon. PON1 activity levels were measured for three substrates—organophosphate paraoxon, arylester phenyl acetate, and lactone dihydrocoumarin—in 767 Mexican American individuals from San Antonio, Texas. Genetic influences on activity levels for each substrate were evaluated by association with approximately one million single nucleotide polymorphism (SNPs) while conditioning on *PON1* genotypes. Significant associations were detected at five loci including regions on chromosomes 4 and 17 known to be associated with atherosclerosis and lipoprotein regulation and loci on chromosome 3 that regulate ubiquitous transcription factors. These loci explain 7.8% of variation in PON1 activity with lactone as a substrate, 5.6% with the arylester, and 3.0% with paraoxon. In light of the potential importance of *PON1* in preventing cardiovascular disease/events, these novel loci merit further investigation.

## 1. Introduction

More than 2,200 Americans die from cardiovascular disease each day with 75% of those deaths attributable to atherosclerosis [1]. Atherosclerosis is characterized by the buildup of fatty lesions, inflammation, and scarring of arterial walls with oxidative stress as a primary contributing factor. Paraoxonase 1 (PON1) is a high-density-lipoprotein-(HDL-) associated esterase which appears to contribute to the antioxidant and antiatherosclerotic capabilities of HDL. PON1 is synthesized in the liver and secreted into the bloodstream where it is capable of breaking down both man-made and naturally occurring compounds. Named for its ability to hydrolyze organophosphates like paraoxon [2, 3] found in insecticides, PON1 is also able to hydrolyze *N-*acyl-homoserine, a lactone used by pathogenic bacteria [4], and lipid peroxides, thereby inhibiting the formation of foam cells known to contribute to atherosclerosis [5, 6].

PON1 has been widely studied following evidence that high activity levels decrease systemic oxidative stress and are associated with a lower incidence of cardiovascular events [7]. PON1 levels have been tied to a number of other disorders including type 1 and 2 diabetes [8, 9], thyroid dysfunction [10], uremia [11], renal failure [12], and inflammatory response [13].

The structural gene *PON1* is by far the largest contributor to variation in serum PON1 activity levels with four known single nucleotide polymorphisms (SNPs) in the promoter region [14–16] and two nonsynonymous substitutions in the coding region of the gene [17, 18] shown to significantly influence activity levels. Amino acid substitution 192Q > R (rs662) specifies 2 allozymes [17] whose differences in activity are substrate dependent. The R allozyme shows greater activity for the organophosphates paraoxon and fenitroxon while the Q form more efficiently hydrolyzes other organophosphates including diazoxon, soman, and sarin. Phenyl acetate is hydrolyzed at the same rate by both forms. [19–21] The 192Q > R substitution is associated with up to 13-fold interindividual differences in PON1 activity [22] and an adjusted hazard ratio for major cardiac events of 1.5 [23]. The 55L > M (rs854560) substitution has also been associated with variation in serum PON1 activity levels, but has a smaller effect size. These polymorphisms have also been linked to Parkinson's disease [24], inflammatory bowel diseases [25], and, controversially, to Alzheimer's disease and vascular dementia [26].

*PON1* is part of a family of genes including *PON2 *and *PON3* located within a 140 kb region at 7q21.3. Although *PON2 *and* PON3* also synthesize paraoxonase proteins, PON2 is not excreted into the blood and any effect by either protein on atherosclerosis or cardiovascular disease is small [27]. Although the *PON* region explains a large degree of the variation in PON1 activity, PON1 activity levels are still better predictors of disease than *PON1* genotypes alone [22, 28]. This supports the existence of additional, unidentified polymorphisms associated with PON1 activity as well as potential epigenetic contributors.

Despite the large body of literature on the *PON* loci, this study is the first to take a genome-wide association approach to identify additional genomic regions contributing to interindividual variation in PON1 activity. Previous studies of this sample identified QTLs for PON1 activity with paraoxon as a substrate on chromosomes 7 (*PON1*), 12, 17, and 19 using whole-genome multipoint linkage analysis [29]. Further investigation, which included alternate substrates and conditioned on the major QTL at chromosome 7, located additional QTLs on chromosomes 1, 3, and 14 [30].

## 2. Methods

### 2.1. Subjects

 Samples were drawn from the San Antonio Family Heart Study (SAFHS) which is composed of 1414 individuals (837 females, 577 males) belonging to 42 extended pedigrees originating with probands randomly ascertained with respect to disease status and phenotype. All probands were Mexican-American individuals between the ages of 40 and 60 at the time of ascertainment, living in San Antonio, TX, with a minimum of six offspring and/or siblings who were at least sixteen years of age and also living in the area. After giving their informed consent, participants underwent a physical examination, demographic and lifestyle interview, and provided blood samples for genotyping and blood chemistry analysis. The study protocol was approved by the Institutional Review Board at the University of Texas Health Science Center in San Antonio and is described in more detail in a previous publication [31].

### 2.2. Paraoxonase Activity

Based on previous evidence of genetic variation giving rise to different activity levels in a substrate-dependent fashion, PON1 activity was assessed on an organophosphate, an arylester, and a lactone. Activity was calculated for 767 individuals based on standard spectrophotometric assays described previously [32]. Briefly, PON1-para activity was determined from the rate of conversion of paraoxon to *p*-nitrophenol, PON1-aryl activity was calculated from the conversion of phenyl acetate to phenol, and PON1-lact activity was based on the conversion of dihydrocoumarin to 3-(2-hydroxyphenyl)propionate. The underlying shared genetic relationship between the activity levels was estimated by calculating the shared genetic covariance (*ρ*_*G*_) for each pair of activity levels. PON-aryl showed significant evidence of a shared genetic contribution with both PON-lact (*ρ*_*G*_ = 0.54, *P* = 1.9*e* − 19) and PON-para (*ρ*_*G*_ = 0.65, *P* = 5.5*e* − 23); however, the genetic correlation between the activity levels for PON-lact and PON-para was essentially zero. This suggests that there are likely to be independent variants influencing activity levels for the different substrates.

### 2.3. Statistical Genetic Analyses

DNA was extracted from buffy coats and used for genotyping on a series of Illumina microarrays (Illumia, Inc., San Diego, CA). 931,219 SNPs passed quality control and were included in the genome-wide association (GWA) analysis. Where it could be done with a high degree of certainty, known pedigree relationships were used to infer missing genotype data using the PEDSYS routine INFER [33]. For ambiguous genotypes, a weighted average of the possible genotypes was used.

Association was assessed for each measurement of paraoxonase activity using the measured genotype test implemented in the program SOLAR (Sequential Oligogenic Linkage Analysis Routines) [34] which takes into account relationships among family members. For all analyses, paraoxonase activity and age were normalized and sex, age, age^2^, and the interaction of sex by age were used as covariates. Additional covariates considered but not found to be associated with PON1 activity in this sample (*P* > 0.1) include dietary measures (proportion of saturated fats, monounsaturated fats, polyunsaturated fats, and fat calories reported in diet), alcohol and cigarette consumption, body mass index (BMI), and total metabolic equivalents as a measure of activity level. To minimize the risk of false associations due to stratification in this admixed sample, principal component analysis was performed on the full set of approximately one million genotypes to capture the total genetic variation in the sample and the first four principal components (accounting for only 2.8% of the variation in the sample) were included as covariates. The efficacy of this correction for stratification was examined by calculating *λ* from the distribution of the lower 90% of *P* values for each GWA. There was no evidence of overdistribution due to stratification as all *λ* values were less than 1.02. Manhattan and Q-Q plots for each GWA can be found in Supplementary File 1 available online at doi:10.1155/2012/189681.

To identify genes contributing to the residual variation, genome-wide association was assessed while including genotypes at the two major *PON1* substitutions 192QR and 55LM (rs662 and rs854560) as covariates. Despite the inclusion of these known variants, other SNPs in the *PON* region of chromosome 7 still showed association with PON1 activity for all three substrates. This suggests that additional variation in *PON1 *or nearby genes is contributing to the variation in PON1 activity levels. To remove all effects of the *PON* loci, four additional SNPs—rs854522, rs854534, rs757158, and rs7803148—each tagging a haploblock in the region surrounding the three *PON* genes—were included as covariates for the GWA analysis. With the inclusion of these covariates, there is 95% power to detect variants with an effect size (*R*_*G*_^2^) of at least 0.041 for PON1-aryl, 0.041 for PON1-lact, and 0.024 for PON1-para.

### 2.4. Candidate Gene Identification

The program SUSPECTS was used to identify candidate genes in the region surrounding each SNP showing significant association with PON1 activity [35]. SUSPECTS determined candidate genes on the basis of similarities in structure, gene ontology, InterPro domains, and/or gene expression with genes known to be related to atherosclerosis, cholesterol regulation, or heart disease. Additionally, all genes within 250 kb of the associated SNP were investigated for potential functional relationships to PON1 activity. A 250 kb region is sufficient to encompass more than 97% of haploblocks in Mexican populations [36]. To further contextualize the results of the GWA analysis, gene set and pathway enrichment analysis was performed using the Database for Annotation, Visualization, and Integrated Discovery (DAVID) [37, 38]. All markers were annotated and the genes found in the top 1% of associations were clustered based on similarity of gene ontology (GO) terms and compared to the genes represented by the remaining 99% of markers.

Additionally, the relationship between gene expression levels measured on an Illumina Sentrix Human Whole Genome (WG-6) Series I BeadChip array [39] and associated SNPs was calculated from the genetic covariance in a polygenic model including sex, age, age^2^, sex*age, 192QR, 55LM, and the additional associated loci in the *PON* region.

## 3. Results

The heritability of paraoxonase activity varies with substrate, *h* ^2^ = 0.65 for phenyl acetate (PON1-aryl), *h* ^2^ = 0.73 for paraoxon (PON1-para), and *h* ^2^ = 0.79 for dihydrocoumarin (PON1-lact) after the inclusion of sex and age in the model. Similarly, the phenotypic variation explained by the covariates and the *PON1* alleles differs among the substrates (Table 1). Cumulatively, age, sex, and their interaction effect explain less than 4% of the variation in PON1 activity for all substrates. In this sample, known *PON1* variants (192QR and 55LM) explain 50.4% of the variation in PON1-para activity but only 9.6% of the variation in PON1-aryl and 12.4% of the variation in PON1-lact activities. Previous research in individuals of Korean ancestry reported that 192QR explained 65.8% of variation in PON1-para activity [40]. A similar study in individuals of European ancestry estimates that 192QR explained 46% and 55LM explained 16% of the PON1-para variation [41]. Although the haplotype blocks in the Mexican American sample are similar in location to those seen in the CEPH Europeans downloaded from HapMap, the linkage disequilibrium (LD) is more extreme, likely due to admixture. This increase in LD would be expected to inflate the explanatory power of the known polymorphisms, so it is unclear why 192QR and 55LM explain less variation in this sample.

To assess the residual variation in the *PON* region of chromosome 7, haplotype blocks were identified using the solid spine of linkage disequilibrium method implemented in Haploview [42]. The SNP rs757158 tags a haploblock including the 5′ promoter region of *PON1 *and explains the greatest proportion of remaining variation in PON1 activity for all substrates. This is in line with previous reports of promoter polymorphisms. As seen for 192QR and 55LM, the promoter polymorphisms have different effects depending on the substrate—this region explains a larger proportion of the variation in PON1-aryl and PON1-lact than in PON1-para. Polymorphisms in the remaining haploblocks are also associated with variation in PON1 activity. rs854534 tags a haplotype block including the majority of the genic region of *PON1*, rs854522 tags a region downstream of all the *PON* genes, and rs7803148 lies in the genic region of *PON2* and tags a haplotype block that includes the entirety of that gene and the majority of *PON3*. Variation in these three haploblocks cumulatively explains less variation in PON1 activity than the haploblock tagged by rs757158. Following the inclusion of these markers as covariates, there are no significant associations in the *PON* regions for PON1-para or PON1-lact; however, PON1-aryl activity is suggestively associated with rs2299262, an intronic SNP in *PON1* that explains 2.8% of the variation in PON1-aryl activity. It should be noted that while the proportion of variation described here and subsequently is useful for comparing the relative contributions of the loci to the variation in activity levels, there can be a substantial upward bias in these estimates unless replicated in an independent sample [43].

The variation in effect size of the polymorphism by substrate bolsters the previously identified substrate-specific effect and indicates additional genetic variants must be contributing to differences in activity levels for PON1-aryl and PON1-lact in particular. The relatively large amount of variation captured by including additional SNPs in the region once the known *PON1 *variants are included in the model suggests finer-scale analysis of this region may identify additional contributing polymorphisms.

Association was assessed for each SNP using a genome-wide significance threshold of *P* < 5*E* − 8 with *P* < 5*E* − 7 considered a suggestive association. As this study assesses associations in Mexican-American families, it should be noted that there is wider linkage disequilibrium than would be found in a population of randomly mating, unadmixed individuals, making this value of *α* conservative. Using these thresholds, two SNPs are associated with PON1-para activity, four with PON1-lact activity (two of which are in perfect LD), and two with PON1-aryl activity after conditioning on the associated SNPs in the *PON* region (Table 2, Supplemental File 1). Only a single SNP (rs1078701) on chromosome 4 is significantly associated with activity on all two substrates and suggestively associated for the third. The proportion of variation explained by rs1078701 varies among the different substrates, ranging from 1.8–2.8%.

Located less than 200 kb from rs1078701, *ACOX3* is a strong positional candidate gene as well as a potential contributor to cholesterol regulation. This gene encodes a peroxisomal pristanoyl-CoA oxidase essential for the catabolism of branched fatty acids into precursors for cholesterol biosynthesis [44]. However, the degree to which peroxisomal fatty acid metabolism contributes to circulating cholesterol is unclear [45]. Also found near rs1078701 is *LRPAP1* which produces a glycoprotein that has been linked with gallstone disease caused by an excess of cholesterol [46] and with cholesterol-related brain disorders such as dementia [47] and Alzheimer's disease [48]. *LRPAP1* regulates the amount of LDL-receptor-related protein expressed in the liver and brain and may also act as a chaperone for lipoprotein lipase [49]. A final candidate gene from this region, *ADD1*, is localized to the erythrocyte membrane and is involved in renal sodium handling and hypertension [50]. It has been implicated in blood pressure, adipogenesis, and coronary heart disease [51, 52]. The other significantly associated SNP is rs7225624 on chromosome 17 which explains 2.9% of the observed variation in PON1-lact. *PCTP* is 6Mbp away from rs7225624, but has a strong SUSPECTS score due the involvement of this gene in cholesterol metabolism and transport as well as lipid binding. PCTP is a transfer protein found in macrophages, which are pervasive in atherosclerotic legions, and work in model organisms indicates *PCTP* regulates lipid efflux into the blood stream [53, 54].

Two additional loci show suggestive associations with PON1 activity. Located on chromosome 1, rs12083993 is associated with variation in PON1-para and replicates a previous linkage result for PON1-aryl [32]. This polymorphism explains 1.2% of the variation in PON1-para and SUSPECTS prioritizes three candidate genes involved in lipid metabolism. *ANGPTL3* is predominantly expressed in the liver, but suppresses lipoprotein lipase in the blood stream which in turn hydrolyzes HDL [55]. Polymorphisms in this gene are also associated with increased arterial wall thickness [56]. *LEPROT* is involved in the cell-surface expression of the leptin receptor, regulation of growth hormones linked to obesity in mice, and cell signaling in response to circulating nutrient levels [57, 58]. Finally, *CYP2J2 *is a member of the cytochrome P450 gene family which is widely involved in the oxidation of organic substances and metabolism. *CYP2J2 *is primarily expressed in the aorta and coronary artery and has been linked to hypertension risk [59, 60]. The most likely mechanism for this relationship is the metabolism of arachidonic acids to epoxyeicosatrienoic acids (EETs) which are vasodilating agents capable of inhibiting inflammatory response and promoting fibrinolysis [61, 62]. Because PON1 can be inactivated under oxidative conditions [63] similar to those present in the absence of functional *CYP2J2* [64, 65], this association may be related to the realized activity of inactivated PON1 enzyme rather than the basal concentration of PON1 or the activity level under normal plasma conditions.

A suggestive association was also found for PON1-lact with two SNPs in perfect LD (rs13322362 and rs11915977) on chromosome 3. Jointly, these explain 2.7% of the overall variation in PON1-lact. Although a region of chromosome 3 was identified in previous linkage analyses [32], that QTL is more than 44 Mbp from rs13322362. This region of the genome contains few genes but may contain one or more transcription factors indicated by the significant association between rs13322362 and transcript levels of three genes including *SLC25A26*, a mitochondrial transport gene [66], *PROK2*, which regulates circadian rhythms [67], and *RYBP*, a broadly expressed binding protein essential for development [68].

The candidate genes identified in this analysis are consistent with the evidence of enrichment of the top 1% of associations for genes analyzed in DAVID. These results suggest the involvement of pathways related to vasculature development and angiogenesis, cell junctions and signaling, cell adhesion, transmembrane glycoproteins and ion transport, and immunoglobulin.

## 4. Discussion

The use of known polymorphisms in *PON1* as covariates in this genome-wide association analysis is an unusual but essential method for identifying regions of the genome with smaller effects. By accounting for more than 40% of the variation in PON1 activity in this way, four additional regions of the genome showing an association with residual PON1 activity were identified that could not otherwise be isolated.

The pathways through which candidate genes influence PON1 activity are, for many of the genes, unclear. Frequently, this is due to a lack of full, functional understanding of the genes themselves. Genes that are associated with cholesterol levels and associated syndromes, but have no clear mechanism, may be contributing to the PON1 pathway but further research would be required to demonstrate this. Because PON1 binds to, and is carried by, nonoxidized HDL molecules, these regions may play a role in plasma HDL concentration which would indirectly increase PON1 concentration and activity. Additionally, it is important to recognize that PON activity is likely influenced by the tertiary structure of the PON1 protein itself or the circulating levels of PON in the blood, which may be influenced by the lipoprotein milieu.

It is necessary, therefore, to consider these results within the broader network of genes and proteins involved in the regulation of lipid metabolism in the blood stream. However, lipid metabolic pathways are not the only ones implicated in these association results. Ubiquitous transcription factors and genes related to oxidative stress were also identified and could play substantial roles in the regulation of PON1 concentration in the blood or the inactivation of PON1 which would decrease activity even in the presence of high levels of the enzyme. Considering the number of factors associated with the activity of single enzyme, future work on the genetic underpinnings and biochemical regulation of atherosclerosis, hypertension, cholesterolemia, and inflammatory diseases more broadly must be understood in the broadest biochemical context.

## Supplementary Material

The supplementary files contain manhattan plots and q-q plots for the results of the genome-wide association studies for PON-aryl, PON-lact, and PON-para activity.

## Figures and Tables

**Table 1:** Proportion of variance explained by covariates. The proportion of variation explained by each significant variable was calculated for the three substrates by adding each to a polygenic model in SOLAR.

	Variation explained		
Covariate	PON1-aryl	PON1-lact	PON1-para
Sex	0.4%	0.0%	0.4%
Age	1.1%	1.4%	0.4%
Age^2^	1.1%	1.9%	0.1%
Sex*age	0.7%	0.4%	0.7%
192QR	3.9%	8.3%	36.5%
55LM	5.7%	4.1%	13.9%
rs854522	2.7%	1.8%	1.1%
rs854534	3.4%	3.8%	2.1%
rs757158	11.5%	9.2%	4.1%
rs7803148	0.9%	1.1%	0.6%

**Table 2:** Summary of Significant and Suggestive Associations. SNP associations varied among substrates with overlap only at rs1078701 on chromosome 4. For each SNP, the chromosomal location, proportion of variation in PON1 activity explained, measured genotype test *P*-value, and minor allele frequency are listed. Shoulder SNPs are SNPs ranking in the top 5% of associations located within 500 kb of the significantly associated SNP. Genes identified by SUSPECTS are within 7.5 Mb of the candidate SNP and have a similarity score greater than 15. Bolded genes are discussed in the texts as the most likely contributors to PON1 activity based on known function, however, other genes may have unknown functions important in the regulation of PON1. The two associated SNPs on chromosome 3 are in perfect linkage disequilibrium and explain 2.7% of the variation in PON1-lact cumulatively.

Substrate	SNP	Chr	Position	Variation Explained	MG* p *	MAF	Shoulder SNPs	SUSPECTS Genes
para	rs12083993	1	64,691,396	1.2%	1.8*E* − 07	0.3%	34	*ALG6, **ANGPTL3**, **CYP2J2**, IL12RB2, INSL5, **LEPROT**, OMA1, PTGER3*
lact	rs13322362	3	76,613,497	2.7%	4.8*E* − 07	5.4%	6	*PROK2, ROBO1, ROBO2*
lact	rs11915977	3	76,613,530		4.8*E* − 07	5.5%		
para				1.8%	4.7*E* − 09		32	
aryl	rs1078701	4	8,241,119	2.8%	3.0*E* − 08	2.2%	19	***ADD1**, CPZ, DGKQ, FGFBP1, FGFR3, FGFRL1, HGFAC, HS3ST1, LRPAP1, MXD4*
lact				2.3%	3.8*E* − 07		15	
aryl	rs2299262	7	94,787,864	2.8%	1.3*E* − 07	43.9%	24	*PON1*
lact	rs7225624	17	47,811,373	2.9%	4.7*E* − 09	0.3%	4	*ABCD4, ADAM11, DGKE, GRN, HOXB2, NGFR, NMT1, OSBPL7, PLCD3, **PCTP**, SCPEP1, SLC35B1*
