# Supplementary material for: Novel Associations of Nonstructural Loci with Paraoxonase Activity
Source: J Lipids. 2012 Apr 17;2012:189681. doi: 10.1155/2012/189681 (PMC3345224; doi:10.1155/2012/189681)
Supplement: Supplementary file 1 — The supplementary files contain manhattan plots and q-q plots for the results of the genome-wide association studies for PON-aryl, PON-lact, and PON-para activity. [file 189681.f1.pdf]

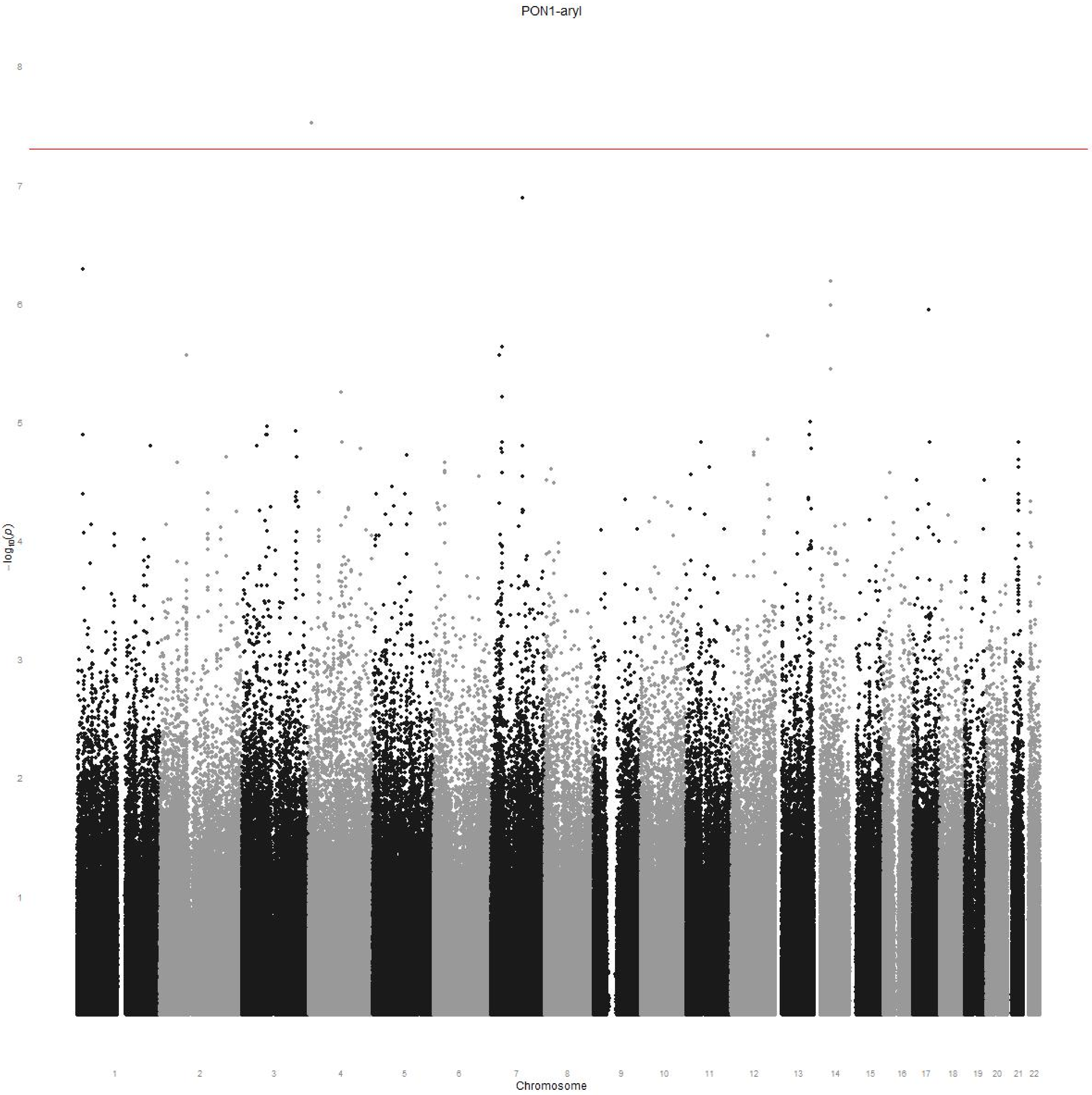

PON1-aryl
8
7
6
5
4
3
2
1
$-\log_{10}(p)$
1
2
3
4
5
6
7
8
9
10
11
12
13
14
15
16
17
18
19
20
21
22
Chromosome

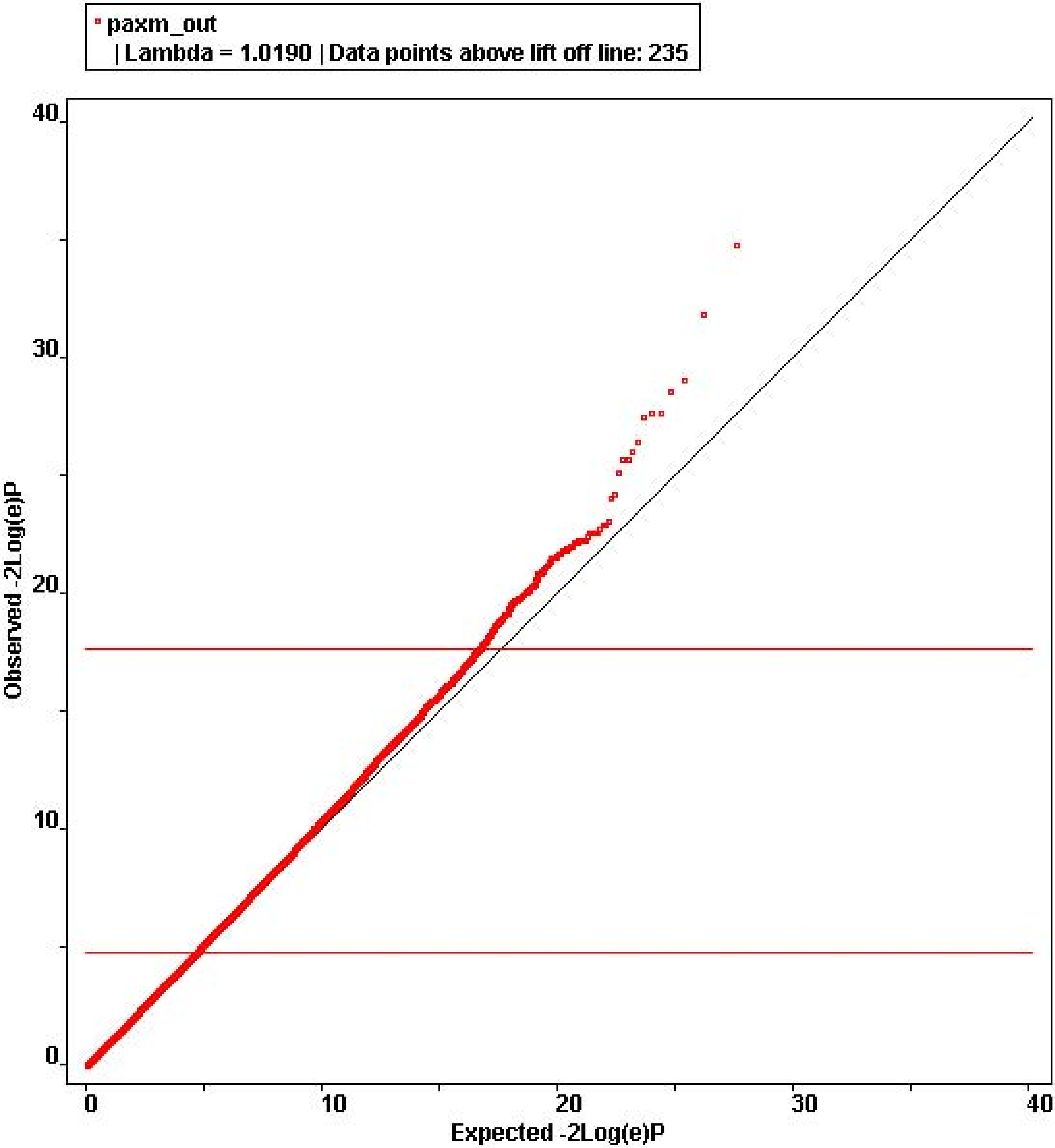

paxm_out
| Lambda = 1.0190 | Data points above lift off line: 235
Observed -2Log(e)P
Expected -2Log(e)P
0
10
20
30
40

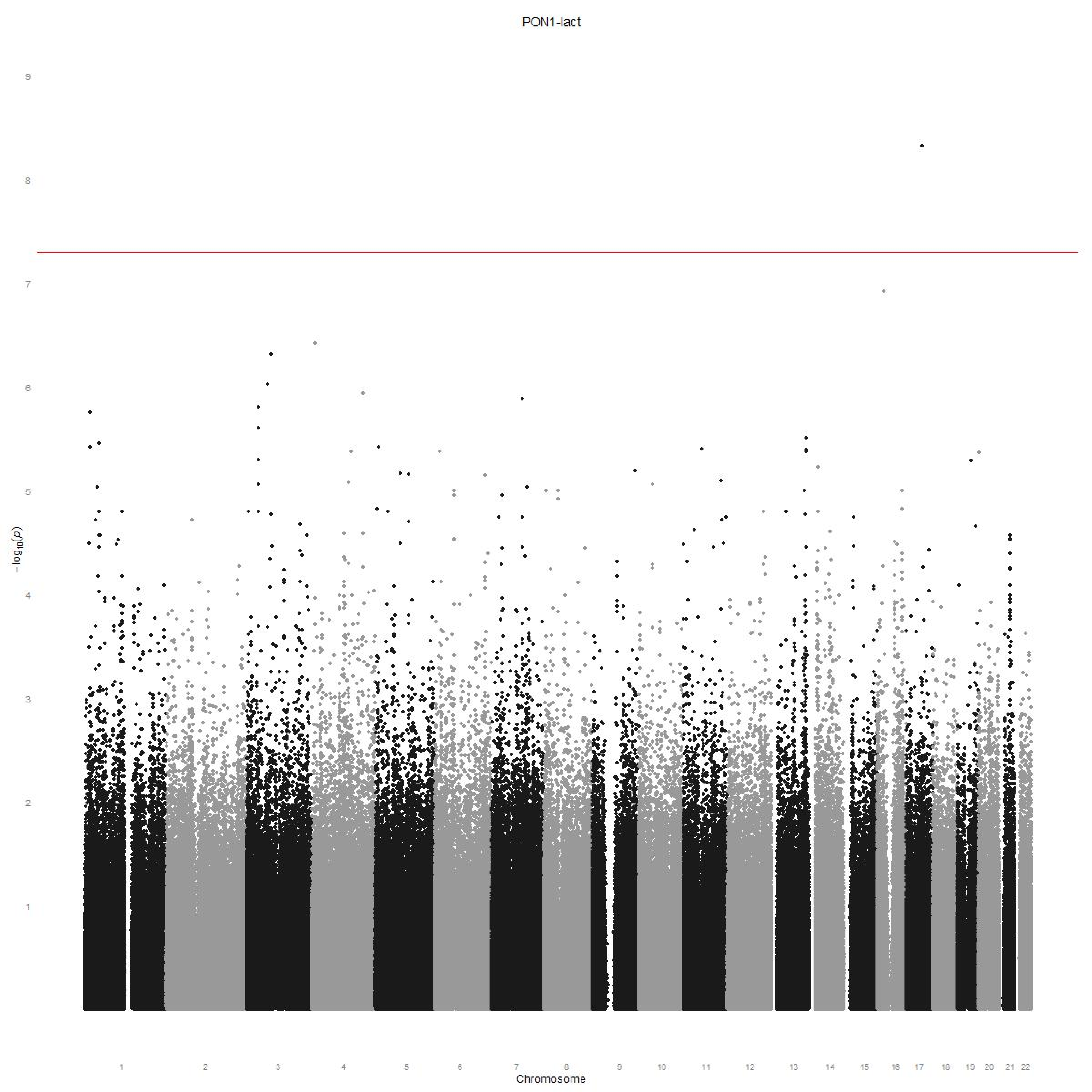
PON1-lact
9
8
7
6
5
4
3
2
1
$-\log_{10}(p)$
1
2
3
4
5
6
7
8
9
10
11
12
13
14
15
16
17
18
19
20
21
22
Chromosome

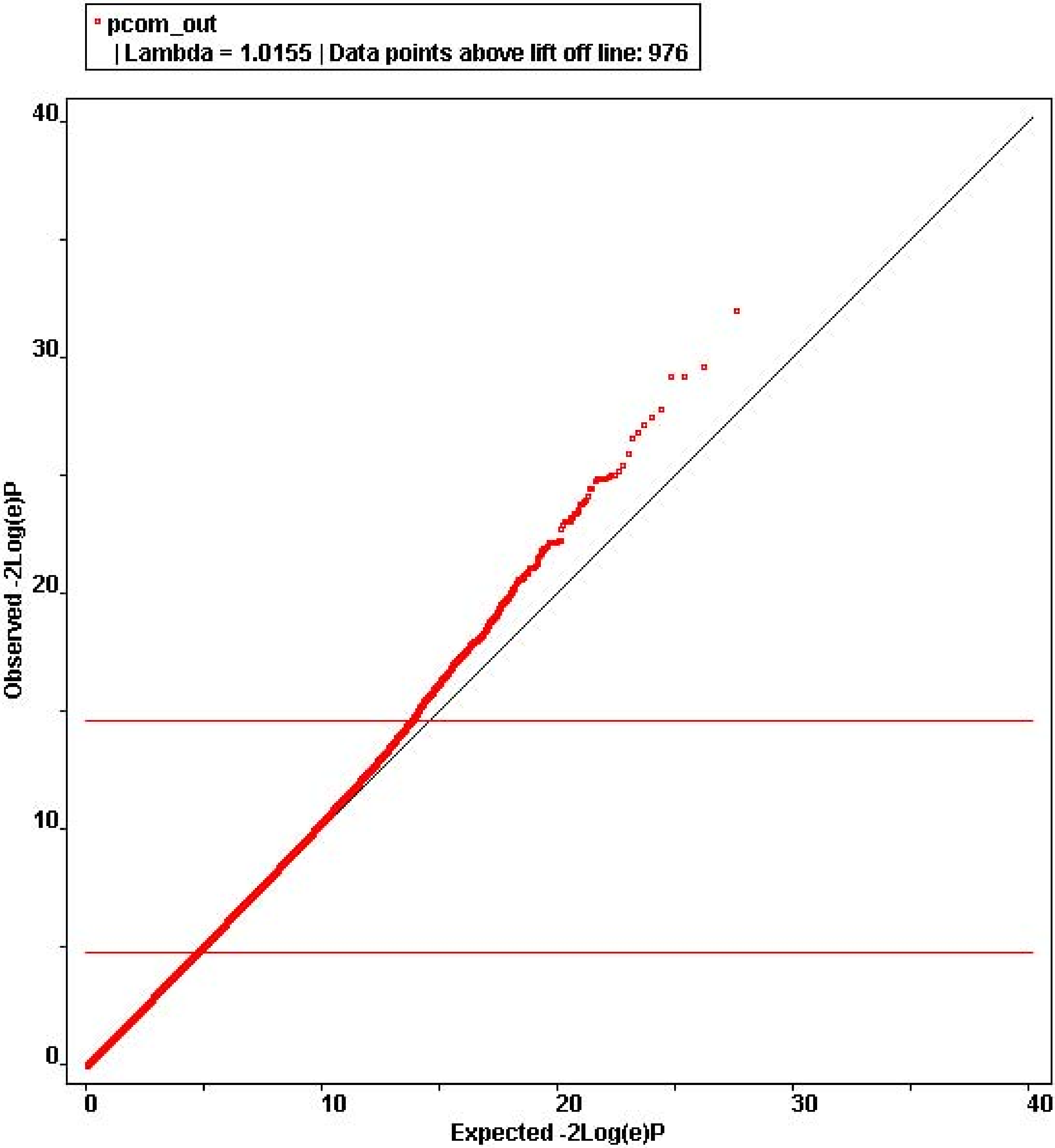
pcom_out
| Lambda = 1.0155 | Data points above lift off line: 976
Observed -2Log(e)P
Expected -2Log(e)P
0
10
20
30
40
0
10
20
30
40

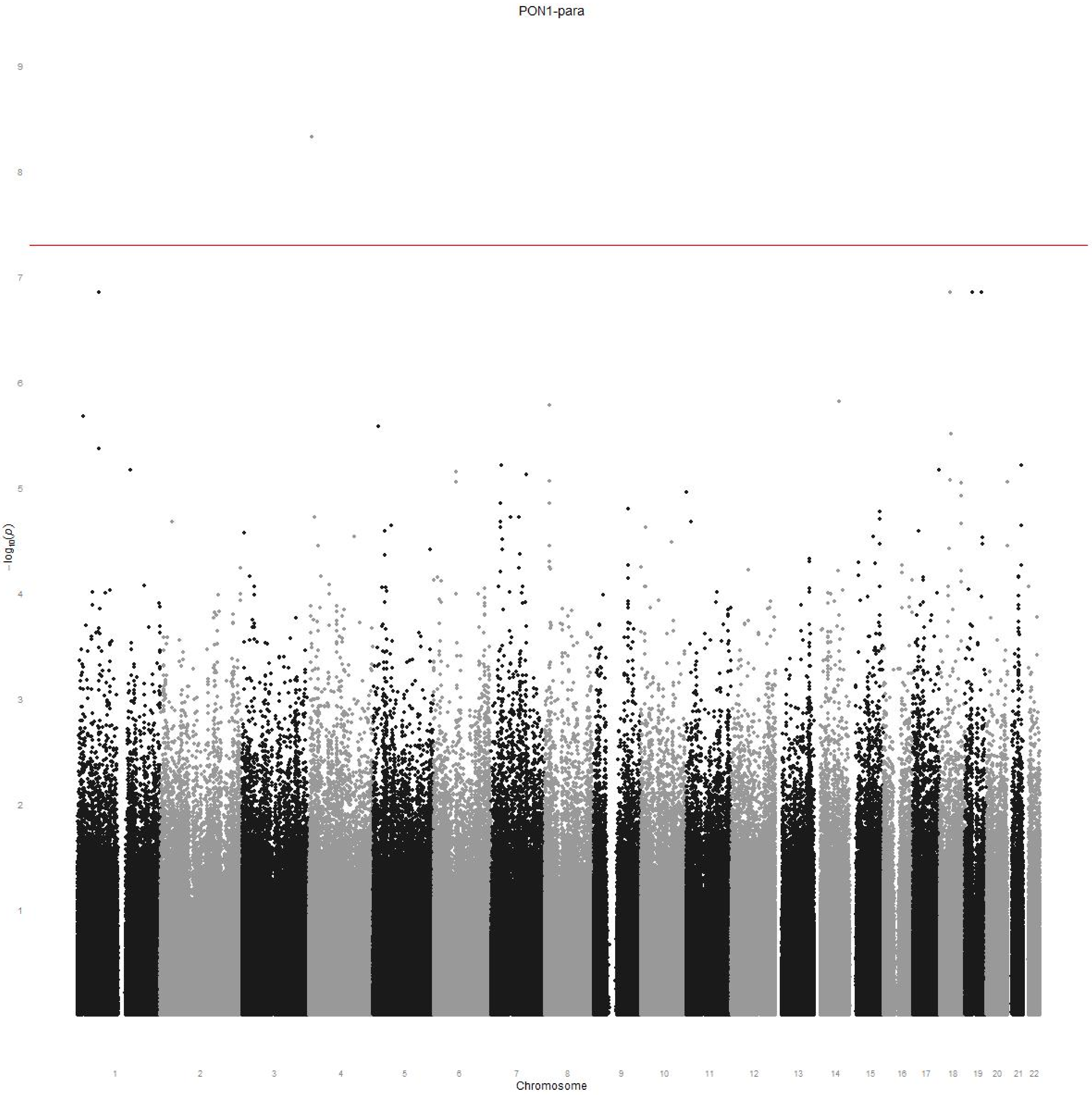

PON1-para
9
8
7
6
5
4
3
2
1
$-\log_{10}(p)$
1
2
3
4
5
6
7
8
9
10
11
12
13
14
15
16
17
18
19
20
21
22
Chromosome

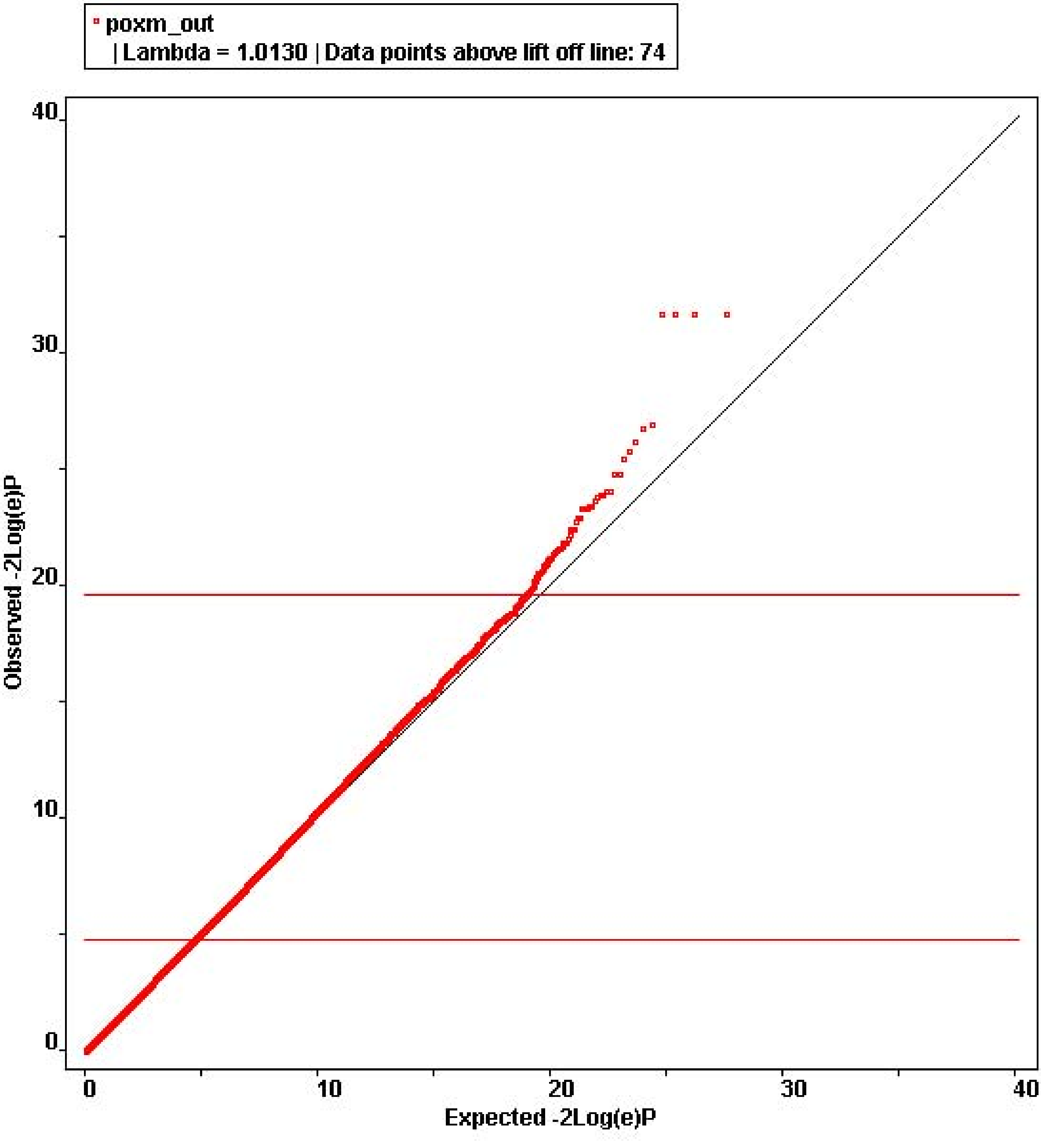

poxm_out
| Lambda = 1.0130 | Data points above lift off line: 74
Observed -2Log(e)P
Expected -2Log(e)P
0
10
20
30
40
